# The Effectiveness of Steroid Versus Vitamin Therapy in the Improvement of Extraocular Movements in Patients With Diabetic Cranial Nerve Palsy: A Comparative Study

**DOI:** 10.7759/cureus.75656

**Published:** 2024-12-13

**Authors:** Inchara N, Smitha Shyam Kumar

**Affiliations:** 1 Ophthalmology, Sri Devaraj Urs Academy of Higher Education and Research, Kolar, IND; 2 Ophthalmology, Laxmi Eye Institute, Mumbai, IND

**Keywords:** challenges, cranial nerve palsy, diabetes, diabetes complications, extraocular movements, mononeuropathy, ophthalmoplegia, steroids, treatment, vitamin therapy

## Abstract

Background: Diabetic mononeuropathies, which are focal neuropathies, are less common than peripheral neuropathy in diabetes. They are frequently underreported or misdiagnosed due to mild or unnoticed cases. Early detection and treatment are crucial to prevent worsening nerve damage and complications.

Objectives: This study aims to compare the effectiveness of conventional treatment with vitamin therapy for improving these neuropathies.

Methods: Fifty cases of diabetic ocular nerve palsy were stratified into two groups, 25 in each. Group A received steroids, while group B received vitamin B12 injections. Ocular movements were assessed before and after treatment initiation at 3, 6, and 10 days using the Kestenbaum limbus test. Inferential statistics were done using the chi-square test and the student t-test.

Results: The average age of the participants in the study was 60.5 ± 6.3 years. Thirty-eight patients had sixth nerve palsy, and 12 patients had associated third nerve palsy. Thirty-eight subjects had poorly controlled diabetes. The mean enhancement in movements was 0.75 ± 0.3 in group A and 1.04 ± 0.5 in group B, with a p value of 0.026, demonstrating the statistically significant difference between both groups.

Conclusion: This study infers that the ocular movements were significantly improved with vitamin therapy. Treating diabetic neuropathy solely with vitamin supplementation indirectly addresses nutritional deficiencies, potentially accelerating diabetes-related complications and enhancing quality of life.

## Introduction

Diabetes mellitus has become a condition of great concern, mainly when associated with poor glycemic control in developing and developed nations. The latest insight given by the diabetes atlas states that the prevalence of diabetes worldwide is currently estimated at 415 million (8.8%) and is expected to rise by another 227 million in the next 25 years [1].

This emerging epidemic, when not under control, inconsiderately affects ocular and systemic complications (acute and chronic), which not only accounts for morbidity but also mortality. Hypo- or hyperglycemia and diabetic coma are acute complications demanding immediate intervention. Chronic complications arise due to micro- and macrovascular changes. Vision-threatening ocular complications of diabetes include diabetic retinopathy, diabetic papillopathy, diabetic neuropathy, glaucoma, cataracts, and ocular surface diseases [2,3].

Neuropathy is a major complication associated with diabetes, leading to significant morbidity among individuals diagnosed with diabetes, particularly within the younger and middle-aged populations. While the majority of patients have distal symmetrical neuropathy, few of them even have focal and multifocal neuropathy, including cranial nerve palsies [4]. Ophthalmoplegia, one of the presentations of neuropathy involving the cranial nerves, is a rare clinical entity and causes great patient anxiety due to sudden diplopia, deviation of the eye to one side, inability to turn the eye to a particular side due to paresis, and ptosis, which makes the patient more worried about the condition. This also poses a serious diagnostic and therapeutic challenge to a clinician in terms of the treatment and prognosis of the disease. Also, there is a lack of evidence for the treatment protocol.

Single or multiple cranial nerve paresis secondary to diabetic neuropathy is a complicated phenomenon affecting the extraocular muscles and presents with variable clinical manifestations [4]. In the literature, diabetic mononeuropathies involving the third or sixth nerve are more frequently encountered than multiple cranial nerve palsies involving the third, sixth, and seventh nerves.

Diabetic oculomotor nerve palsies typically present with diplopia and ptosis, with pupillary function being spared [5,6]. However, this condition is known to be self-limiting with strict diabetic control; complete recovery may take six weeks to six months. Therefore, to hasten the process, in many studies, steroids and multivitamin injections have been given. Concerning the above literature, in this study, we attempted to compare the effect of systemic steroids versus intravenous vitamin B12 injection in diabetic cranial nerve palsy. Also, we compared the incidence of single and multiple cranial nerve palsies in people with diabetes.

## Materials and methods

Study overview

After giving their written informed consent, 50 patients attending the Ophthalmology OPD with ocular neuropathy due to diabetes mellitus were included in the study. A complete history of diabetes, the onset of the ocular problem, and its duration were noted. All the patients underwent thorough ocular examination, including visual acuity by Snellen chart, pupillary shape, size, and reaction, and slit lamp examination, including dilated fundus evaluation with +90D and indirect ophthalmoscopy. Extraocular movements were measured with the help of the Kestenbaum limbus test. Qualitative and quantitative squint assessments were done for all the patients, including motor and sensory tests.

Ethical considerations

A written consent form, which mentioned the complete information about the study, confidentiality, and compensation, was taken before recruiting the subjects into the study. Approval from the Institutional Ethics Committee from Sri Devaraj Urs Academy of Higher Education and Research, letter no. SDUMC/KLR/IEC/106/2019-20, dated November 26, 2019, was obtained prior to commencing the study.

Sample size calculation

Over one year, we received 50 cases of diabetic cranial nerve palsy. These cases were randomly divided into two groups, each receiving a different treatment, allowing us to compare outcomes and assess the effectiveness of each treatment approach.

Study criteria

Patients with unilateral ocular motor nerve paresis or paralysis due to diabetes were included in the study. Patients with a history of head injury or trauma, congenital strabismus, history of neurosurgery, intracranial pathologies, and other existing systemic comorbidities such as asthma and cardiac conditions were excluded.

Abducens (VI) nerve palsy was considered if the patient had binocular horizontal diplopia and esotropia in the primary gaze with a deviation of the eye nasally on the affected side. The oculomotor (III) cranial nerve palsy was characterized by one or more signs: droopy lid, restricted upward ocular motility, and binocular diplopia. The trochlear (IV) cranial nerve palsy was considered if the patient presented with vertical diplopia, commonly accompanied by compensatory contra lateral head tilt [6,7].

CT scan and MRI of the brain and or orbit were done in doubtful cases like ocular nerve palsy in young patients with diabetes and patients with nonlocalizing symptoms like headache, projectile vomiting, and painful ocular movements if they coexisted with proptosis to rule out orbital apex syndrome or even cavernous sinus syndrome. These patients were excluded from the study and were referred to a neurologist/neurosurgeon as they needed emergency intervention.

All 50 patients underwent blood investigations, such as complete blood count, random blood sugar, fasting blood sugar, postprandial blood sugar, hemoglobin A1c, lipid profile, blood urea, and creatinine. Physician opinions were taken for all cases to evaluate diabetes status systemically and achieve strict glycemic control.

Procedure

These patients were categorized into two groups. Group A received systemic steroids: the steroids given were Tab methylprednisolone, once a day (1 mg/kg of body weight), and tapered over 10 days. Group B received vitamin injection: vitamin injection included a single injection consisting of a combination of water-soluble B vitamins (thiamine 10 mg, riboflavin 10 mg, nicotinamide 45 mg, calcium pantothenate 50 mg, pyridoxine 3 mg, and cobalamin 15 mcg) given intramuscularly on alternate days, a total of five doses over 10 days.

During the hospital stay, patients were put on a diabetic diet and medications (oral and systemic) as opined by the treating physician for attaining strict glycemic control with three times a day monitoring of glucometer random blood sugar. Lifestyle modification was encouraged.

Assessments

Extraocular movements were measured in each patient on days 3, 6, and 10 starting the treatment. Differences in the deviation and improvement in the excursion of ocular movements were measured using a ruler using the same Kestenbaum limbus test (Kestenbaum limbus test: reference values in the healthy population are 5-7 mm on elevation and 9-10 mm on depression, adduction, and abduction) [8,9]. Then, the pre- and posttreatment measurements of the extraocular movements in both groups were compared.

Statistical analysis

Descriptive statistics was done with proportions and mean ± SD, and inferential statistics was done by tests of significance, namely, the chi-square test and the Student t-test, where p < 0.05 was considered statistically significant.

## Results

A total of 50 patients were included in the study. The mean age of the study participants is 60.5 ± 6.3 years, with the mean age among group A at 60.2 ± 6.7 years and group B at 60.9 ± 5.9 years (Table 1). There were 17 male and eight female patients in group A, and nine male and 16 female patients in group B, which concludes that the incidence of cranial nerve palsy was slightly higher in men than women but statistically insignificant (Table 1). Poor glycemic control with an HbA1c range of 8.1%-10 gm% was commonly seen in 21 patients, and an HbA1c of more than 10.1 gm% was seen in 14 patients. Thirty-eight patients of the total had sixth nerve palsy, and 12 patients had associated third nerve palsy (Table 1).

**Table 1:** Distribution of study participants in both groups (n = 50) DM: diabetes mellitus; HTN: hypertension; NPDR: nonproliferative diabetic retinopathy

Variable		Group A (n = 25), n (%)	Group B (n = 25), n (%)	Total, n (%)	p value
Gender	Male	17 (68%)	16 (64%)	33 (66%)	0.765
	Female	8 (32%)	9 (36%)	17 (34%)	
Age (in years)	41-50	2 (8%)	1 (4%)	3 (6%)	0.209
	51-60	13 (52%)	5 (20%)	18 (36%)	
	>60	10 (40%)	19 (76%)	29 (58%)	
Systemic comorbidities	DM alone	15 (60%)	13 (52%)	28 (56%)	0.568
	DM + HTN	10 (40%)	12 (52%)	22 (44%)	
Fundus examination, right eye	Normal	22 (88%)	16 (64%)	38 (76%)	0.252
	Mild NPDR	1 (4%)	4 (16%)	5 (10%)	
	Moderate NPDR	1 (4%)	3 (12%)	4 (8%)	
	Mixed retinopathy	1 (4%)	2 (8%)	3 (6%)	
Fundus examination, left eye	Normal	22 (88%)	16 (64%)	38 (76%)	0.252
	Mild NPDR	1 (4%)	4 (16%)	5 (10%)	
	Moderate NPDR	1 (4%)	3 (12%)	4 (8%)	
	Mixed retinopathy	1 (4%)	2 (4%)	3 (6%)	
HbA1C levels	<6 gm%	2 (8%)	1 (4%)	3 (6%)	0.821
	6.1-8 gm%	10 (40%)	12 (48%)	22 (44%)	
	8.1-10 gm%	11 (44%)	9 (36%)	20 (40%)	
	>10.1 gm%	2 (8%)	3 (12%)	5 (10%)	

The subjects in group B had (better) improvement in the excursion of the ocular movements (abduction, adduction, elevation, and depression) compared to those in group A, which is statistically significant (Table 2). The mean improvement in the movements, 0.75 and 1.04 in groups A and B, respectively, again proves the statistically significant improvement in group B (Table 3).

**Table 2:** Comparison of effectiveness of the therapy in both groups (n = 25 in each group) ^*^p < 0.05 is statistically significant

Variable		Group A (n = 25), n (%)	Group B (n = 25), n (%)	Total, n (%)	p value
Improvement in extraocular movements	0.0-0.5 mm	8 (32%)	2 (8%)	10 (20%)	0.029^*^
	0.6-1.0 mm	10 (40%)	6 (24%)	16 (32%)	
	1.1-1.5 mm	1 (4%)	4 (16%)	5 (10%)	
	1.6-2.0 mm	6 (24%)	13 (52%)	19 (38%)	
Improvement in ptosis	0.0-0.5 mm	3 (12%)	5 (20%)	8 (16%)	0.127
	0.6-1.5 mm	15 (60%)	11 (44%)	26 (52%)	
	1.6-2.0 mm	1 (4%)	6 (24%)	7 (14%)	
	2.0-2.5 mm	6 (24%)	3 (12%)	9 (18%)	

**Table 3:** Improvement in extraocular movements in both groups (n = 25 in each group) ^*^p < 0.05 is statistically significant

Variable	Group A (n = 25)	Group B (n = 25)	t value	p value
Mean improvement in extraocular movements	0.72 ± 0.3	1.04 ± 0.5	2.2879	0.026^*^

## Discussion

The predicament of diabetes control demonstrates a positive relationship to ocular motor nerve palsy cases. Only three out of 50 patients had well-managed diabetes at the time of presentation, while the vast majority had moderate, poor, or inferior control when they first presented.

Although the information on diabetes management before the presentation was gathered through patient history, it provided an insight into how diabetes was controlled. This information was then compared with findings from similar studies conducted locally and in countries with similar socioeconomic conditions, such as Saudi Arabia and Iran [10,11]. It appears that poor management of diabetes is more common, leading to a higher incidence of diabetic sixth nerve palsy compared to other causes. In Western countries, diabetic sixth nerve palsy is not always the most frequent cause, which suggests better overall diabetes control in the general population [12-15].

The Diabetes Control and Complications Trial (DCCT) demonstrated that better blood glucose control can lower the risk of developing diabetic polyneuropathy (DPN) and autonomic neuropathy in patients with diabetes. The DCCT findings strongly indicate that maintaining optimal blood glucose levels can help prevent DPN and autonomic neuropathy in people with diabetes. No other studies have shown that modifying other risk factors effectively prevents diabetic neuropathies [14-16].

One of the most frequent complications of type 2 diabetes mellitus is peripheral neuropathy, and its development has been associated with vitamin B12 and folic acid deficiencies, brought about by hyperhomocysteinemia in subjects with diabetes. A study relating vitamins to type 2 diabetes has concluded that supplementation of vitamin B12 will help prevent diabetes complications like neuropathy. Recent reports state that antidiabetic medications like metformin cause vitamin B12 deficiency, which makes a good point for the rationale of this study. Pyridoxine (B6) has a positive effect on the vascular complication of diabetes, and thiamine (B1) showed an impact on the glucose tolerance curve, making another point toward validating the trial of this combination of vitamins in sixth nerve palsy in cases of isolated diabetes [17-22].

The effect of combined vitamin B therapy on the outcome of diabetic isolated abducent nerve palsy

In our study, we encountered 50 cases of diabetic ophthalmoplegia, divided into two groups of 25 patients each. Group A had 25 patients for whom only an oral steroid tapering dose was administered. For group B patients, we put them on vitamin B complex with vitamin B12 injection of five on alternative days. Improvement was noted early and significantly in group B, as they were on combination therapy, and the results were statistically significant.

Diabetic ophthalmoplegia typically occurs in patients with diabetes over the age of 50, affecting both type 1 and type 2 diabetes. The onset is usually rapid, developing within a day or two. Many patients experience pain for a few hours to a few days before noticing double vision (diplopia). In a study by Green et al. [23], pain preceded the onset of diplopia in 14 out of 25 patients. Pain is more commonly reported when the third cranial nerve is affected than the sixth nerve. The pain is generally aching and located behind or above the eye, sometimes more diffuse, but always on the same side as the oculomotor palsy. This pain is often thought to result from the involvement of the first and second divisions of the trigeminal nerve within the cavernous sinus or from the activation of pain-sensitive endings within the sheath of the third nerve as it passes through the cavernous sinus. Pain usually does not persist after the onset of diplopia. Oculomotor dysfunction tends to be incomplete when the third nerve only affects one or two muscles, leading to partial paralysis [24-27].

In their study of 22 episodes of ophthalmoplegia in 20 patients, Goldstein and Cogan [28] reported 12 episodes of complete dysfunction, three episodes of nearly complete dysfunction, and three cases of partial paralysis. Ptosis is often pronounced, and the eye deviates outward when the internal rectus muscle is affected. The patient cannot move the eye medially, upward, or downward. The pupillary function is typically preserved, which helps distinguish third nerve palsy of diabetic origin from that caused by compression due to an aneurysm of the posterior communicating artery, where pupillary dilation is common. Spontaneous and complete recovery usually occurs within two to three months, regardless of the level of hyperglycemia control.

Proximal diabetic neuropathy is frequently very painful and often does not respond to conventional treatments. In such cases, corticosteroid treatment can be considered for a few weeks or months, with adjustments made to diabetes management. Not all patients with proximal diabetic neuropathy require treatment, as spontaneous recovery can occur, even when inflammatory lesions are present on nerve biopsy specimens. However, we believe corticosteroid treatment is essential when multiple nerve lesions are present [29,30]. It is suggested in the literature to start with prednisone 0.75 mg/kg body weight/day for three to four weeks, then taper gently. Diabetes control must be adjusted after the introduction of corticosteroids. However, the addition of steroids is likely to disturb diabetes control in most patients. So, in this study, steroids have been complimented with vitamin injections to bring in a multidisciplinary approach [30-32]. Though no study proves the treatment of diabetic neuropathy with vitamin supplementation alone, it is an indirect effort in treating the nutritional deficiencies to hasten the complications of diabetes and improve the quality of life. Often, ophthalmoplegia improves without treatment, but relapses occur.

Strengths and limitations

In our study, subjects were quantitatively evaluated using the Kestenbaum limbus test, and patients were continuously monitored up to the 14th day after receiving injections. However, the study's accuracy could have been improved by including a control group receiving no treatment or a placebo. Additionally, the lack of long-term follow-up with these patients prevented a thorough assessment of the effects of vitamin therapy.

## Conclusions

Early diagnosis and prompt management of ocular diabetic ophthalmoplegia are essential to prevent long-term complications following paralysis and to promote faster recovery from the condition. Regular screening for vitamin deficiencies in patients with diabetes should be part of their routine care to identify and address any potential contributors to the development of ophthalmoplegia. Maintaining reasonable glycemic control remains fundamental in preventing microvascular complications associated with diabetes. This study suggests incorporating strategies to address vitamin deficiencies, whether directly or indirectly linked to diabetes, should also be included in the treatment plan. Additionally, it is vital for patients with diabetes to be aware of this potential complication, which can cause sudden ocular symptoms, and to be reassured that a good recovery is achievable with proper metabolic control and vitamin supplementation.
